# Impact of home monitoring program on interstage mortality after the Norwood procedure

**DOI:** 10.3389/fcvm.2023.1239477

**Published:** 2023-10-11

**Authors:** Helena Staehler, Thibault Schaeffer, Johanna Wasner, Julia Lemmer, Michel Adam, Melchior Burri, Alfred Hager, Peter Ewert, Jürgen Hörer, Masamichi Ono, Paul Philipp Heinisch

**Affiliations:** ^1^ Department of Congenital and Pediatric Heart Surgery, German Heart Center Munich, Technische Universität München, Munich, Germany; ^2^ Division of Congenital and Pediatric Heart Surgery, University Hospital of Munich, Ludwig-Maximilians-Universität, Munich, Germany; ^3^ Department of Congenital Heart Disease and Pediatric Cardiology, German Heart Center Munich, Technische Universität München, Munich, Germany; ^4^ Department of Cardiovascular Surgery, German Heart Center Munich, Technische Universität München, Munich, Germany

**Keywords:** hypoplastic left heart syndrome, Norwood procedure, interstage home monitoring program, bidirectional cavopulmonary shunt, BCPS

## Abstract

**Objective:**

While early outcome after the Norwood operation for hypoplastic left heart syndrome has improved, interstage mortality until bidirectional cavopulmonary shunt (BCPS) remains a concern. Our aim was to institute a home monitoring program to (HMP) decrease interstage mortality.

**Methods:**

Among 264 patients who survived Norwood procedure and were discharged before BCPS, 80 patients were included in the HMP and compared to the remaining 184 patients regarding interstage mortality. In patients with HMP, events during the interstage period were evaluated.

**Results:**

Interstage mortality was 8% (*n* = 21), and was significantly lower in patients with HMP (2.5%, *n* = 2), compared to those without (10.3%, *n* = 19, *p* = 0.031). Patients with interstage mortality had significantly lower birth weight (*p* < 0.001) compared to those without. Lower birth weight (*p* < 0.001), extra corporeal membrane oxygenation support (*p* = 0.002), and lack of HMP (*p* = 0.048) were risk factors for interstage mortality. Most frequent event during home monitoring was low saturation (<70%) in 14 patients (18%), followed by infection in 6 (7.5%), stagnated weight gain in 5 (6.3%), hypoxic shock in 3 (3.8%) and arrhythmias in 2 (2.5%). An unexpected readmission was needed in 24 patients (30%). In those patients, age (*p* = 0.001) and weight at BCPS (*p* = 0.007) were significantly lower compared to those without readmission, but the survival after BCPS was comparable between the groups.

**Conclusions:**

Interstage HMP permits timely intervention and led to an important decrease in interstage mortality. One-third of the patients with home monitoring program needed re-admission and demonstrated the need for earlier stage 2 palliation.

## Introduction

The neonatal Norwood procedure for hypoplastic left heart syndrome (HLHS) and its variant has undergone constant improvement and modification to archive best possible results (1, 2). Nevertheless, the crucial phase does not end when patients are discharged from hospital but spans the interstage period. Various research has indicated that interstage mortality may range from 2% to as much as 20% (3–5). Risk factors for adverse events during that period have been studied throughout the years. Several factors, such as low birth weight, anatomical subtypes, or extra-cardiac anomalies, are associated with higher interstage mortality rates. However, due to their inherent characteristics, these factors are mostly beyond the reach of operative interventions (4, 6, 7). Therefore, patients affected by these factors require close monitoring (6).

Frequent complications that may arise include excessive hypoxemia, hypovolemia, change in systemic and pulmonary vascular resistance or progressive myocardial dysfunction (5, 8, 9). Particularly, stenosis of modified Blalock-Taussig shunt (MBTS) or right ventricle to pulmonary artery conduit (RVPAC) may disturb the balance between systemic and pulmonary circulations (9). Respiratory illnesses might also be a risk for interstage complications. Hence, our aim was to identify potentially critical courses before patients would present with fulminant complications. To extend the monitoring of key parameters beyond hospital discharge, we launched a home monitoring program (HMP) according to the report of Ghanayem, et al. (10). HMP aims at detecting critical changes, such as drop of oxygen saturation and decrease in weight gain. In addition, HMP serves to support parents and custodians in the care of critically ill children. Many studies have presented positive results in reducing interstage mortality by implementation of similarly structured programmes (9, 10). Rudd et al. have reported excellent interstage survival rates of 98% following the introduction of their HMP (11).

This study intends to evaluate the following two points: (1) whether introduction of HMP reduced the interstage mortality after the Norwood procedure. (2) How HMP detected the problems of patients, and how HMP influenced the outcomes after bidirectional cavopulmonary shunt (BCPS).

## Patients and methods

### Ethical statement

This study was approved by the Institutional Review Board of the Technical University of Munich (approved number of 305/20 S-KH on 2 June, 2020). Because of its retrospective nature, the need for individual patient consent was waived.

### Patients

**Figure 1:**
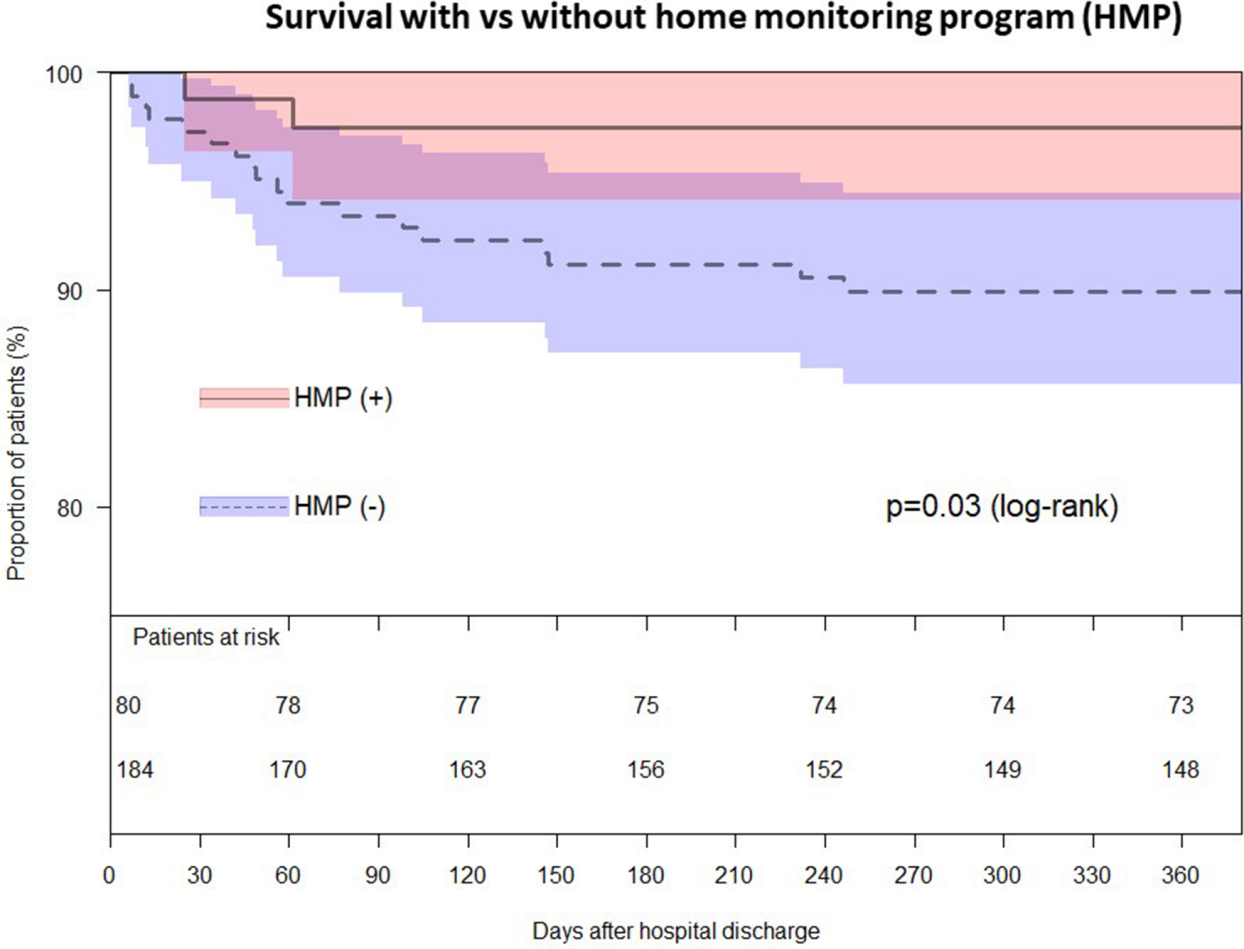
Flow chart of patients undergoing the Norwood procedure.

Between January 2001 and December 2020, 335 consecutive patients with HLHS and its variants underwent the neonatal Norwood procedure at the German Heart Centre of Munich. Patients who were subsequently discharged from hospital with shunt-dependent physiology were included in this study (*n* = 264). Patients who died in hospital and those who underwent BCPS following S1P without being discharged in the interim were excluded from the study (Figure 1). Pre- and postoperative data was obtained by the review of the patients' medical records. Since 2013, patients with single ventricle physiology were included in a home monitoring program (*n* = 80) which allowed the assessment of adverse events prior to BCPS and if so, therefrom resulting interstage mortality.

### Operative techniques

The Norwood procedure is performed under standard cardiopulmonary bypass (CPB) with hypothermic circulatory arrest. Selective cerebral perfusion has been performed since 2009. The details of the operative techniques are described in our previous reports (12, 13). The choice of shunt was made at the discretion of patients’ surgeon and cardiologist. In patients who received RVPAC, a 5.0 mm ringed Gore-Tex graft was used in most patients. In patients who underwent MBTS, most patients received a 3.5 mm non-ringed Gore-Tex graft. All patients were postoperatively admitted to the intensive care unit (ICU) with chest open. Delayed sternal closure was performed usually on the second postoperative day.

### Home monitoring program

In 2013, an interstage HMP was established at our center, which allowed close monitoring and regular check-ups of the patients. The HMP team consists of a nurse case manager and a cardiologist (J.L.), who offers additional support. During the implementation phase, the personnel involved were exclusively sourced from the existing resources within the Division of Pediatric Cardiology. Over time, there emerged a need for the administration to allocate time towards team communication, acquire equipment, and maintain the program. The suppliers of durable medical equipment supplied pulse oximeters, primarily receiving reimbursement from patients' insurance after the HMP team submitted a letter of medical necessity. Patients' caretakers were instructed in measuring and documenting the patient's daily percutaneous oxygen saturation (SpO2), heart rate, weight gain and feeding protocol at least twice a week. Surveillance criteria were measured using pulse oximeters and sensitive digital infant scales. Parents were informed that resting SpO2 less than 70%, weight loss of 30 g or failure to gain 20 g of weight for 3 days were to be considered critical and that in the respective case a call to the hospital would have to be made (12). Furthermore, parents received the usual discharge information to recognize warning signs for respiratory or gastrointestinal illness, respiratory distress, such as tachypnea and nasal flaring, or changes in perfusion. If problems arose, any necessary action was decided upon depending on the severity of the symptoms.

### Identification of factors affecting mortality

Interstage mortality, defined as mortality between hospital discharge and BCPS, was analyzed using Cox-regression model. Pre-, intra- and postoperative variables were analyzed as risk factors.

### Follow-up data

With the exception of the Hospital Management Program (HMP), all patients received outpatient follow-up care from pediatric cardiologists. The survey concluded for patients who experienced mortality by recording the time of their death as the endpoint. The cause of death was not ascertainable for patients who experienced death outside of a healthcare facility.

The subsequent data were consistently monitored and revised utilizing our institutional database system specifically designed for single-ventricle cases.

In our imaging laboratories, the grading of aortic valve regurgitation (AVVR) was performed using transthoracic echocardiography, which follows established principles that entail the semiquantitative assessment of AVVR through the utilization of Doppler color flow. This evaluation is based on the assessment of the ratio between the area of the regurgitant jet and the area of the atrium. A regurgitant color jet area-to-atrial area of less than 30% signifies mild aortic valve regurgitation, while a range of 30%–50% indicates moderate regurgitation, and a value exceeding 50% indicates severe aortic valve regurgitation.

### Statistical analysis

Categorical variables are presented as absolute numbers and percentages. Continuous variables are expressed as medians with interquartile ranges (IQR). An independent Student´s *t*-test was used to compare normally distributed variables. The Mann-Whitney *U* test was used for variables that were not normally distributed. Survival after hospital discharge and survival after BCPS was estimated by the Kaplan-Meier method. Risk factors for mortality were identified using uni- and multi-variate Cox regression models. Weight fo age *z*-score (WAZ) was calculated using the WHO Anthro software 3.2. Data analysis was performed using SPSS version 25.0 for Windows (IBM, Ehningen, Germany) and R statistical software 4.2.1 (R Foundation for Statistical Computing).

## Results

### Patients and outcomes after the Norwood procedure

Patientś characteristics are shown in Table 1. Median birth weight was 3.2 (2.9–3.5) kg and 16 (6.3%) patients had a birth weight of less than 2.5 kg. Main diagnosis included 217 HLHS (82.2%) and 47 variants (17.8%). In 217 patients with HLHS, anatomic sub-types included 50 aortic atresia (AA)/mitral atresia (MA), 57 AA/mitral stenosis (MS), 26 aortic stenosis (AS)/MA and 88 AS/MS. Genetic anomalies and extra-cardiac anomalies were observed in 11 (4.3%) and in 30 patients (11.8%), respectively. Birth weight was significantly lower in patients with interstage deaths compared to those without interstage death (*p* < 0.001). Other variables were similar between the groups.

**Table 1:** Patient characteristics.

Variables	Total	Interstage survivals	Interstage deaths	*p* value
	*n* = 264	243 (92)	21 (8)	
Male gender	182 (68.9)	169 (69.5)	13 (61.9)	0.468
Gestational age (weeks)	39 (38–40)	39 (28–40)	39 (37–39)	0.201
Birth weight (kg)	3.2 (2.9–3.5)	3.3 (2.9–3.6)	3.0 (2.2–3.2)	<0.001
Low birth weight (<2.5 kg)	16 (6.3)	13 (5.5)	3 (16.7)	0.06
Genetic anomalies	11 (4.3)	11 (4.7)	0 (0)	0.32
Extracardiac anomalies	30 (11.8)	27 (11.5)	3 (15.0)	0.64
EFE	25 (9.7)	22 (9.3)	3 (14.3)	0.46
HLHS	217 (82.2)	200 (82.3)	17 (81.0)	0.877
AA/MA	50 (22.6)	46 (22.5)	4 (23.5)	0.926
AA/MS	57 (25.9)	52 (25.6)	5 (29.4)	0.731
AS/MA	26 (11.8)	24 (11.8)	2 (11.8)	0.994
AS/MS	88 (40.0)	82 (40.4)	6 (35.3)	0.680
Variant	47 (17.8)	43 (17.7)	4 (19.0)	0.877

EFE, endocardial fibroelastosis; HLHS, hypoplastic left heart syndrome; AA, aortic atresia; MA, mitral atresia; AS, aortic stenosis; MS, mitral stenosis.

Operative and postoperative data are shown in Table 2. Median age and weight at the Norwood procedure were 9 (7–12) days and 3.2 (2.9–3.5) kg, respectively. As for shunt-types, MBTS was performed in 133 (50.4%) patients, and RVPAC in 131 (49.6%). Median age and weight at hospital discharge were 24 (16–35) days and 3.2 (3.0–3.7) kg, respectively. Patients with interstage death had lower weight at Norwood (*p* = 0.008), longer CPB time (*p* = 0.033), and higher incidence of extracorporeal membrane oxygen (ECMO) support (*p* < 0.001), compared to those without.

**Table 2:** Operative- and Post-operative variables.

Variables	Total	Interstage survivals	Interstage deaths	*p* value
	*n* = 264	243 (92)	21 (8)	
Age at Norwood	9 (7–12)	9 (7–11)	9 (7–13)	0.112
Weight at Norwood	3.2 (2.9–3.5)	3.2 (3.0–3.5)	3.1 (2.8–3.3)	**0** **.** **008**
CPB time	137 (104–159)	134 (102–159)	138 (122–175)	**0**.**033**
AXC time	48 (40–58)	48 (40–58)	46 (41–57)	0.388
RVPAC	131 (49.6)	124 (51.0)	7 (33.3)	0.120
Intubation	5 (4–8)	5 (4–7)	4 (3–11)	0.120
ICU stay	12 (8–20)	12 (8–19)	13 (7–13)	0.065
ECMO	11 (4.2)	7 (2.9)	4 (19.0)	**<0**.**001**
Re-OP after Norwood	68 (26.4)	61 (25.7)	7 (33.3)	0.449
Re-intubation	40 (15.6)	36 (15.3)	4 (19.0)	0.652
Shunt intervention	27 (10.4)	24 (10.0)	3 (14.3)	0.541
Re CoA intervention	9 (3.5)	8 (3.3)	1 (4.8)	0.734
Peritoneal dialysis	18 (7.0)	15 (6.4)	3 (14.3)	0.175
NEC	14 (5.5)	11 (4.7)	3 (14.3)	0.065
Length of hospital stay	24 (16–35)	24 (16–35)	22 (15–61)	0.149
Age at discharge	35 (27–46)	35 (27–46)	36 (27–71)	0.069
Weight at discharge	3.2 (3.0–3.7)	3.3 (3.0–3.7)	3.1 (2.9–3.7)	0.184
SO2 at discharge	85 (80–87)	85 (80–87)	85 (80–85)	0.156
HMP possible	80 (30.3)	78 (32.1)	2 (9.5)	**0**.**031**

HMP, home monitoring program; AA, aortic atresia; MA, mitral atresia; AS, aortic stenosis; MS, mitral stenosis.

The bold values in the analysis indicate the most statistically significant results.

### Interstage mortality

**Figure 2:**
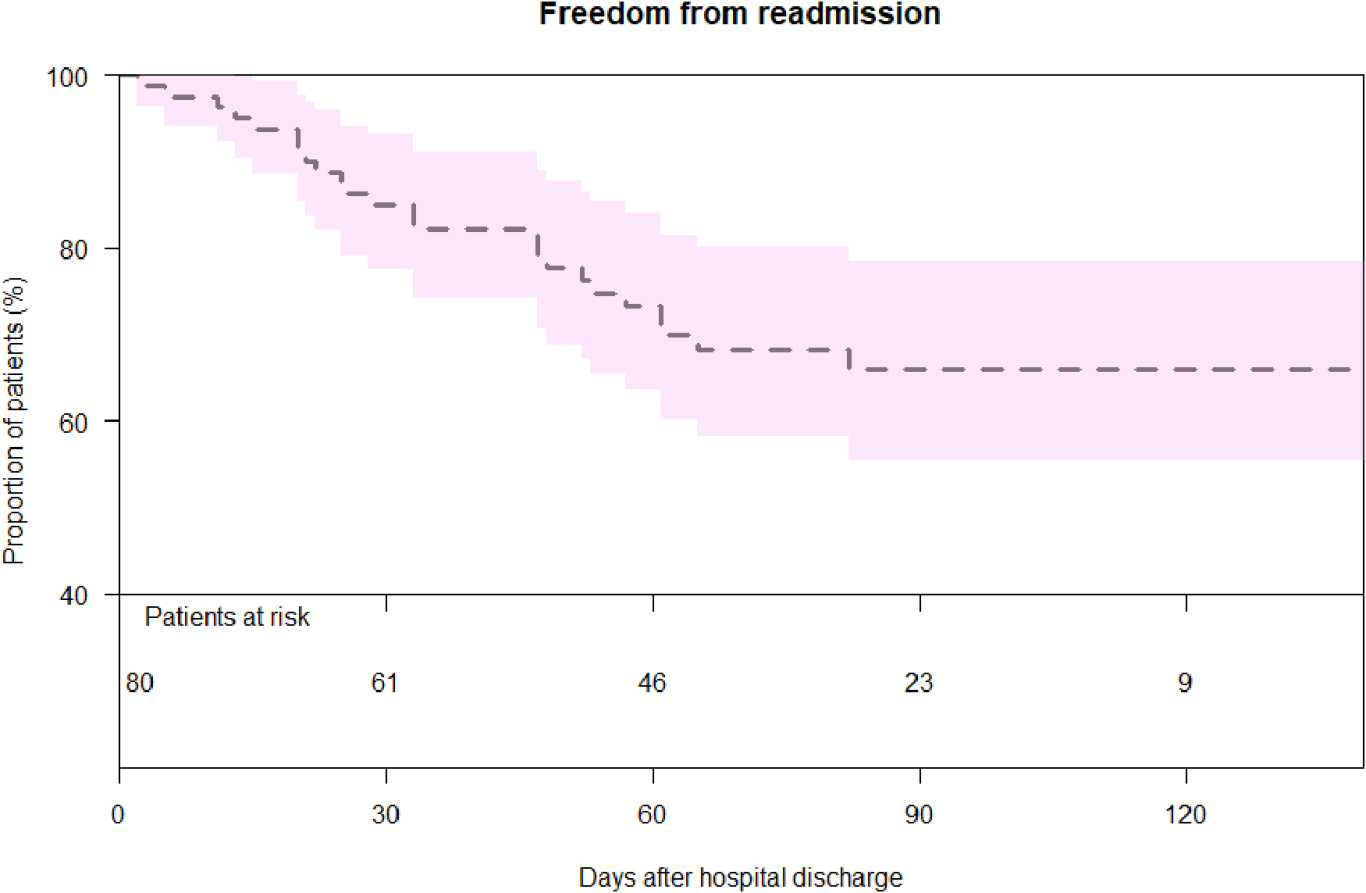
Survival after hospital discharge in patients with and without HMP.

A total of 21 cases of interstage mortality were observed during the period between hospital discharge and the next stage of the BCPS procedure. The cause of deaths were 12 cases of low-cardiac-output, 4 sudden deaths, 2 progressive hypoxias, and 3 unclear causes of death. Since the implementation of the Hospital Management Program (HMP) in our institution in 2013, a total of 80 patients have been enrolled (Figure 2). Nevertheless, a cohort comprising 71 individuals waere excluded from receiving HMP treatment due to either their lack of residency in Germany or their inability to consistently provide updates on their health status for diverse reasons. The interstage mortality of patients who were included into the HMP (*n* = 2, 2.5%) was lower than the mortality of patients without HMP (*n* = 19, 11.6%, *p* = 0.031). Risk factors for interstage death are shown in Table 3. Lower birth weight (*p* < 0.001), weight at Norwood (*p* = 0.016), longer ICU stay (*p* = 0.005), need for ECMO support (*p* = 0.002) and lack of HMP (*p* = 0.048) were identified as risk factors using univariable analysis. Multivariable analysis identified lower birth weight (*p* = 0.003) as an independent risk factor. When we selected 151 patients who had undergone the Norwood procedure since 2013 and performed the same analysis, weight at Norwood (*p* = 0.008), need for ECMO (*p* < 0.001) and lack of HMP (*p* < 0.001) were identified as independent risk factors (Supplementary Table S1).

**Table 3:** Analysis of candidate risk factors for interstage mortality following the Norwood procedure.

Variable	Univariable model			Multivariable model		
	HR	(95% CI)	*p* value	HR	(95% CI)	*p* value
Birth related variables						
Premature birth						
Birth weight (per gram)	0.572	0.411–0.795	<0.001	0.998	0.997–0.999	0.003
Genetic anomalies	0.046	0.000–542.300	0.521			
Extracardiac anomalies	1.299	0.381–4.433	0.676			
HLHS	0.899	0.302–2.672	0.848			
AA	1.517	0.644–3.573	0.340			
MA	1.410	0.555–3.581	0.470			
UAVSD	1.500	0.201–11.179	0.692			
Reduced VF						
AVVR pre Norwood	0.042	0.000–29.638	0.344			
Restrictive ASD	0.979	0.395–2.425	0.963			
Ao Asc. diameter	1.025	0.823–1.277	0.827			
Norwood variables						
Age at Norwood	1.048	0.974–1.128	0.210			
Weight at Norwood	0.300	0.112–0.800	0.016			
RVPAC	0.486	0.196–1.203	0.119			
Postoperative variables						
Intubation	1.025	0.985–1.066	0.220			
ICU stay	1.018	1.005–1.031	0.005			
HSP stay	1.011	0.997–1.025	0.132			
ECMO	5.756	1.936–17.113	0.002	4.158	0.939–18.407	0.061
Re OP	1.393	0.562–3.452	0.474			
Re Intubation	1.332	0.448–3.960	0.606			
PD	2.146	0.632–7.286	0.221			
NEC	3.140	0.925–10.664	0.067			
Shunt intervention	1.407	0.414–4.777	0.584			
Re-CoA intervention	1.387	0.186–10.337	0.749			
Findings at discharge						
Weight	1.000	0.999–1.001	0.349			
Systolic BP	0.966	0.920–1.015	0.176			
Diastoolic BP	0.960	0.900–1.024	0.217			
Mean BP	0.918	0.812–1.039	0.178			
SO2	0.942	0.836–1.061	0.327			
Heart rate	0.976	0.935–1.018	0.256			
HMP (-)	4.347	1.014–18.867	0.048	3.460	0.771–14.705	0.103

### Results of HMP

**Figure 3:**
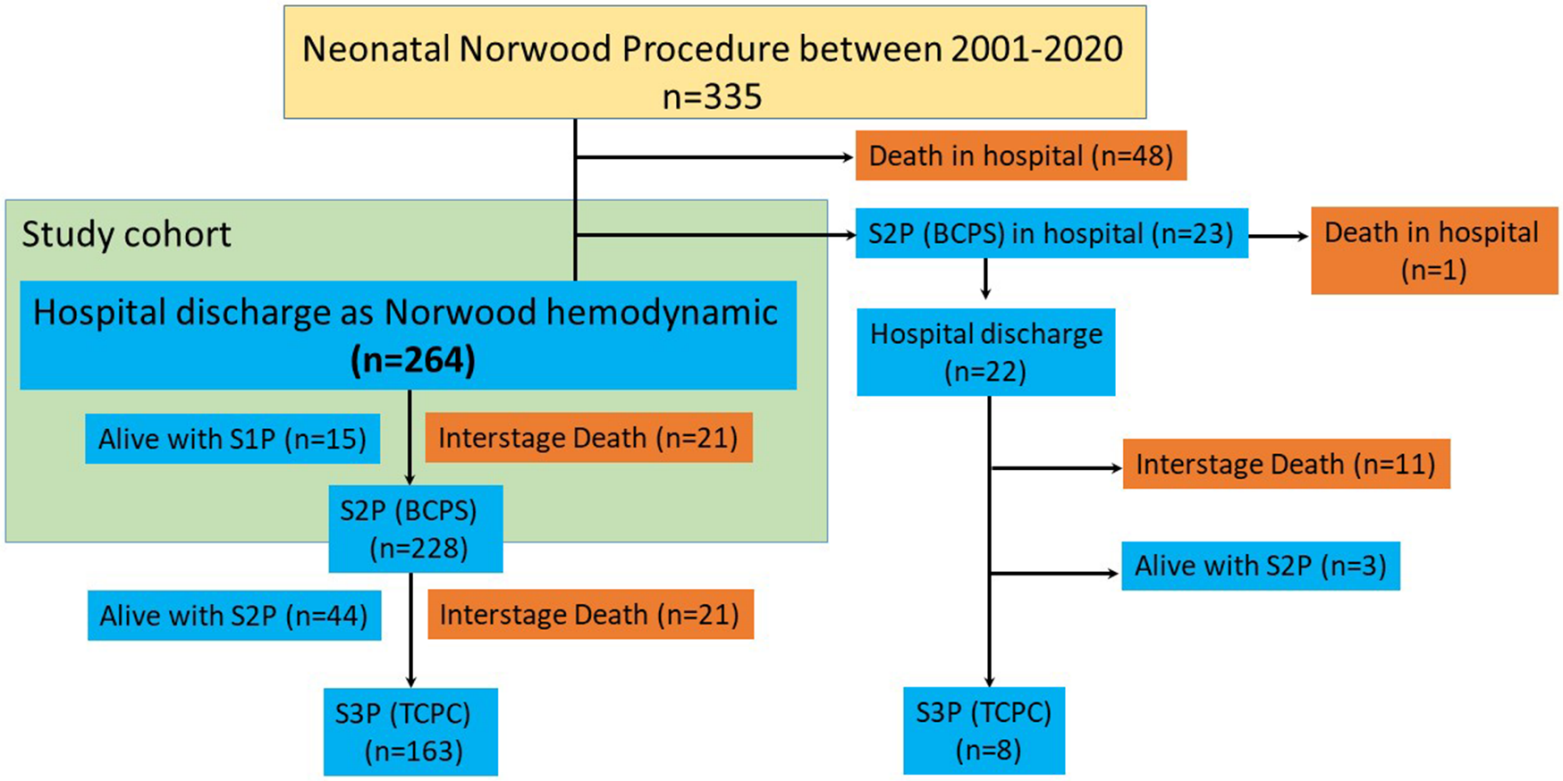
Freedom from readmission after hospital discharge.

The events that occurred within the HMP during interstage period are shown in Table 4. The most frequent event was low saturation (<70%) in 14 patients (18%), followed by infection (*n* = 6, 7.5%), stagnated weight gain (*n* = 5, 6.3%), hypoxic shock (*n* = 3, 3.8%), arrhythmia (*n* = 2, 2.5%) and side effects of vaccinations (*n* = 2, 2.5%). Readmission to the hospital was required in 24 (30%) patients with a median 29 (18–54) days after the hospital discharge (Figure 3). No variable at hospital discharge was identified as a risk for readmission (Supplementary Table S2). Shunt-types showed no difference in the incidence of readmission (Supplementary Figure S1).

**Table 4:** Events of Home Monitoring Program (HMP).

Events	*N* (%) or median (IQR)
HMP patient number	80
Low SO2 <70%	14 (17,5)
Arrhythmia	2 (2.5)
Infection	6 (7.5)
Vaccination effect	2 (2.5)
Hypoxic shock	3 (3.8)
Stagnated weight gain	5 (6.3)
Readmission	24 (30.0)
Interval (days)	29 (18–54)

### Characteristics at the time of BCPS

Characteristics at the time of BCPS in patients with and without HMP are shown in Table 5A. Age at BCPS (*p* = 0.015), and Z score of weight at BCPS (*p* = 0.010) were significantly lower in patients with HMP. Mortality after BCPS was similar between the groups (*p* = 0.500). In patients with HMP, the patient characteristics at the time of BCPS with and without readmission are shown in Table 5B. Age at BCPS (*p* = 0.001) and weight at BCPS (*p* = 0.007) were lower in patients who needed readmission. However, mortality after BCPS was similar between patients who needed readmission and those who did not (*p* = 0.614).

**Table 5:** Characteristics at time of BCPS.

A. Patients with and without HMP				
Variables	All	HMP (+)	HMP (-)	*p*-value
*N*	228	157 (85.3)	71 (88.8)	0.456
Age at BCPS (months)	4.0 (3.2–5.0)	3.7 (3.0–4.6)	4.1 (3.3–5.3)	**0** **.** **015**
Weight at BCPS (kg)	5.1 (4.5–5.9)	5.0 (4.6–5.6)	5.2 (4.5–6.0)	0.298
Weight Z score	1.9 (2.7–1.1)	1.7 (2.4–0.9)	2.1 (2.8–1.2)	**0**.**010**
Mortality after BCPS	21 (8.0)	16 (8.7)	5 (6.3)	0.500
B. Patients with and without readmission during interstage				
Variables	All	Re admission (+)	Re admission (-)	*p*-value
*N*	71	22 (91.7)	49 (87.5)	0.589
Age at BCPS (months)	3.7 (3.0–4.6)	3.1 (2.8–3.7)	3.9 (3.4–4.8)	**0**.**001**
Weight at BCPS (kg)	5.0 (4.6–5.6)	4.7 (4.3–5.1)	5.2 (4.8–5.9)	**0**.**007**
Weight Z score	1.7 (2.4–0.9)	1.8 (2.6–1.3)	1.7 (2.4–0.7)	0.135
Mortality after BCPS	5 (6.3)	2 (8.3)	3 (5.4)	0.614

The bold values in the analysis indicate the most statistically significant results.

### Comment

Interstage mortality was lower in patients with HMP compared to those without. Lower birth weight, ECMO support after the Norwood procedure, and lack of HMP were risks for interstage mortality. Most frequent events during home monitoring were low percutaneous oxygen saturation below 70%, infection and stagnated weight gain. One-third of the patients needed unintended readmission. Age and weight at BCPS were lower in patients who needed readmission compared to those who did not. However, survival after BCPS was similar between the groups with and without readmission.

### Interstage mortality after the Norwood procedure

Interstage mortality accounts for an increasing proportion of mortality after the Norwood procedure. Most of interstage mortality occurred in patients with shunt dependent physiology between stage I and stage II palliations. Incidence of interstage mortality is reported to be up to 12% in a multi-center trial (4). Previous studies have identified several pre- and postoperative variables as risk factors for interstage mortality (13–16).

### Home monitoring program

In 2013, our facility launched an interstage HMP, which enabled more thorough patient monitoring as well as more frequent check-ins. The unintended benefits were earlier recognition of inadequate growth and the elimination of growth failure that is commonly reported in infants with shunt-dependent heart disease (11). During the interstage period, 30% of patients were re-admitted for observation without cases of cardiac shock. Interstage mortality was reduced significantly from 12.4% to 2.2% after the introduction of the HMP. Two mortalities under HMP occurred in its early era (one patient in 2013 and another in 2014). Since 2015, we experienced no interstage mortality in patients who were supported by HMP. When compared to previous single- and multicenter reports, this represents a significant drop in overall mortality as well as morbidity rate between stages (9, 17–19).

In addition, our findings demonstrate that the introduction of HMP decreases interstage mortality in patients undergoing the Norwood procedure. The mortality rate decreased significantly throughout the staged intervention, cutting down from 12.4% to 2.2%. After the start of HMP, a significantly higher number of patients was unable to be discharged prior to BCPS (12/57 vs. 8/105, *p* = 0.022). This directly resulted from the implementation of HMP. At the time of BCPS, the children who had been part of the HMP were significantly younger and significantly lighter than those who were part of the remainder. At that time, their ages ranged from 67 to 299 days. After BCPS, early survival rate was not affected in any discernible way.

HMP identifies physiologic disturbances before decompensation and provides an early warning system for prompt interventions. This resulted in a significant reduction of interstage mortality in our population. 31% (*n* = 14) of the patients needed interstage treatment because of HMP concerns and of these, 8 patients required early surgical procedures.

In accordance with previous publications, we matched our study participants to a historic control group. Therefore, additional confounding factors, such as developments in care over the past 19 years, may account for the reported outcomes (9, 10, 20). However, we believe that surgical modifications and the improvement of care protocols are more likely to influence the early postoperative period. The most important factors affecting the interstage period, including imbalanced systemic and pulmonary circulation, congestive heart failure, the risk of shunt stenosis, and recurrent aortic arch stenosis, are comparable amongst the study groups (20).

### Refining of the HMP and future prospective

Hansen et al. implicated that the thorough training of parents before discharge is crucial to improve their understanding of potential problems their child might encounter at home and to emphasize the importance of using the monitor continuously in situations where they are not directly interacting with the child (9). The HMP began with desired thresholds for SpO2 and weight change. The programme expanded to include circulatory insufficiency and failure to thrive based on initial findings, patient outcomes and parental feedback. Several programming enhancements were implemented concurrently to increase the efficiency of this home surveillance technology. Before discharge, dietary targets, standard equipment, systematic data-recording, educational materials, and room-in parent care were set. Imaging and lab data are collected during interstage clinic visits only when clinically necessary. Time required for pre-S2P echocardiography and cardiac catheterization varies by patients. The effectiveness of the HMP has been improved, in our opinion, by conducting consultations over the phone. The parents took advantage of additional counselling by phone, and the consistency of therapy gave the families additional confidence.

These findings do not indicate whether improved survival is due to more precise measurement of physiologic variables, improved care coordination, or both. The HMP is a complex care paradigm that improves communication between families and healthcare providers. These newborns are cared for at home by a multidisciplinary team of clinicians who work closely together. In our experience, there was a lower threshold for contact between parents and HMP NPs as a result of the surveillance calls. Furthermore, coordination between community cardiologists and the surgical site was improved, as was the transition from inpatient to outpatient treatment. When a local cardiologist or pediatrician is unavailable, the HMP provides an additional resource for the family or emergency medical personnel. The Norwood procedure with HMP is a model with far-reaching implications for the care of children with chronic conditions outside of the hospital, enabling multidisciplinary care coordination across pediatric disciplines.

### Study limitations

All studies are based on observational, non-randomized data from a single center, limiting the study's ability to be generalized. We examined the study group to a historical control group, so any confounding variables, such as advances in supportive care over 19 years, could potentially account for the observed result. In addition, recent patients in the control group were in part coming from abroad, some of them from regions, where medical service is not as developed as in Germany. Thirdly, some patients were in a too bad condition to be discharged until BCPS and could not go into HMP. We had insufficient information regarding critical events in the control group throughout the interstage period, and only parents or local physicians reported the cause of death. None of the infants were subjected to post-mortem examinations.

## Conclusions

The home monitoring programme, which allowed for timely intervention, primarily early stage 2 palliation, reduced interstage mortality significantly. A greater number of patients remained hospitalized until the bidirectional cavopulmonary shunt was installed as a result of adhering to the surveillance criteria prior to discharge. In the surveillance group, earlier BCPS had no negative effect on early survival after bidirectional cavopulmonary shunt.

## Data Availability

The raw data supporting the conclusions of this article will be made available by the authors, without undue reservation.

## Supplementary material

Supplementary Figure S1 Freedom from readmission after hospital discharge in patients with MBTS and with RVPAC.
