# Supplementary material for: Impact of home monitoring program on interstage mortality after the Norwood procedure
Source: Front Cardiovasc Med. 2023 Oct 11;10:1239477. doi: 10.3389/fcvm.2023.1239477 (PMC10600023; doi:10.3389/fcvm.2023.1239477)
Supplement: Supplementary file 1 [file Datasheet1.docx]

**Supplementary File**

Supplementary Table S1

| Table S1. Analysis of candidate risk factors for inter-stage mortality following the Norwood procedure | | | | | | | |
| --- | --- | --- | --- | --- | --- | --- | --- |
| Variable | Univariable model | | |  | Multivariable model | | |
|  | HR | (95% CI) | p value |  | HR | (95% CI) | p value |
| **Birth related variables** | |  |  |  |  |  |  |
| Premature birth | 0.935 | 0.365-2.395 | 0.888 |  |  |  |  |
| Birth weight (per kilogram) | 0.752 | 0.587-0.965 | 0.025 |  |  |  |  |
| Genetic anomalies | 1.402 | 0.192-10.220 | 0.739 |  |  |  |  |
| Extracardiac anomalies | 2.632 | 1.096-6.318 | 0.030 |  |  |  |  |
| HLHS | 1.179 | 0.520-2.671 | 0.694 |  |  |  |  |
| AA | 2.271 | 1.167-4.421 | 0.016 |  |  |  |  |
| MA | 1.932 | 1.030-3.622 | 0.040 |  |  |  |  |
| UAVSD | 0.567 | 0.078-4.134 | 0.576 |  |  |  |  |
| Reduced VF | 0.305 | 0.042-2.223 | 0.241 |  |  |  |  |
| AVVR pre Norwood | 1.024 | 0.744-1.410 | 0.882 |  |  |  |  |
| Restrictive ASD | 3.367 | 1.728-6.560 | <0.001 |  |  |  |  |
| Ao Asc. diameter | 0.816 | 0.679-0.980 | 0.030 |  |  |  |  |
| **Norwood related variables** | |  |  |  |  |  |  |
| Age at Norwood | 0.977 | 0.912-1.048 | 0.519 |  |  |  |  |
| Weight at Norwood | 0.296 | 0.56-0.561 | <0.001 |  | 0.377 | 0.183-0.774 | 0.008 |
| RVPAC | 1.383 | 0.737-2.595 | 0.313 |  |  |  |  |
| **Postoperative variables** | |  |  |  |  |  |  |
| Intubation | 1.058 | 1.035-1.082 | <0.001 |  |  |  |  |
| ICU stay | 1.007 | 0.997-1.018 | 0.164 |  |  |  |  |
| HSP stay | 0.991 | 0.979-1.003 | 0.137 |  |  |  |  |
| ECMO | 10.661 | 5.419-20.971 | <0.001 |  | 4.900 | 2.313-10.383 | <0.001 |
| Re OP | 3.802 | 1.987-7.276 | <0.001 |  |  |  |  |
| Re Intubation | 0.941 | 0.394-2.246 | 0.891 |  |  |  |  |
| PD | 2.539 | 1.284-5.020 | 0.007 |  |  |  |  |
| NEC | 2.143 | 1.017-4.519 | 0.045 |  |  |  |  |
| Shunt intervention | 2.636 | 1.389-5.005 | 0.003 |  |  |  |  |
| Re-CoA intervention | 3.465 | 1.636-7.341 | 0.001 |  |  |  |  |
| **Findings at discharge** | |  |  |  |  |  |  |
| Weight | 0.999 | 0.998-1.000 | 0.056 |  |  |  |  |
| Systolic BP | 0.973 | 0.910-1.040 | 0.416 |  |  |  |  |
| Diastoolic BP | 0.953 | 0.884-1.027 | 0.203 |  |  |  |  |
| Mean BP | 0.895 | 0.769-1.040 | 0.148 |  |  |  |  |
| SO2 | 1.021 | 0.873-1.194 | 0.794 |  |  |  |  |
| Heart rate | 0.961 | 0.910-1.015 | 0.156 |  |  |  |  |
| HMP (-) | 30.303 | 7.246-125.000 | <0.001 |  | 15.625 | 3.558-66.667 | <0.001 |

Supplementary Table 2.

| Table S2. Risk factor for re-admission in 80 patients with HMP | | | |
| --- | --- | --- | --- |
| Variables | HR | 95% CI | p-value |
| **Findings at discharge** | |  |  |
| Weight | 1.000 | 0.999-1.001 | 0.892 |
| Systolic BP | 1.005 | 0.960-1.052 | 0.833 |
| Diastoolic BP | 0.987 | 0.947-1.029 | 0.540 |
| Mean BP | 1.021 | 0.945-1.103 | 0.603 |
| SO2 | 0.987 | 0.874-1.115 | 0.837 |
| Heart rate | 1.033 | 0.986-1.081 | 0.170 |
| **Other variables** |  |  |  |
| Birth weight | 0.848 | 0.484-1.484 | 0.563 |
| Extracardiac anomalies | 0.048 | 0.000-13953 | 0.636 |
| Restrictive ASD | 1.004 | 0.430-2.346 | 0.993 |
| Weight at Norwood | 1.128 | 0.473-2.691 | 0.786 |
| ECMO | 0.048 | 0.000-3134 | 0.591 |

**Supplementary Figure S1**

**
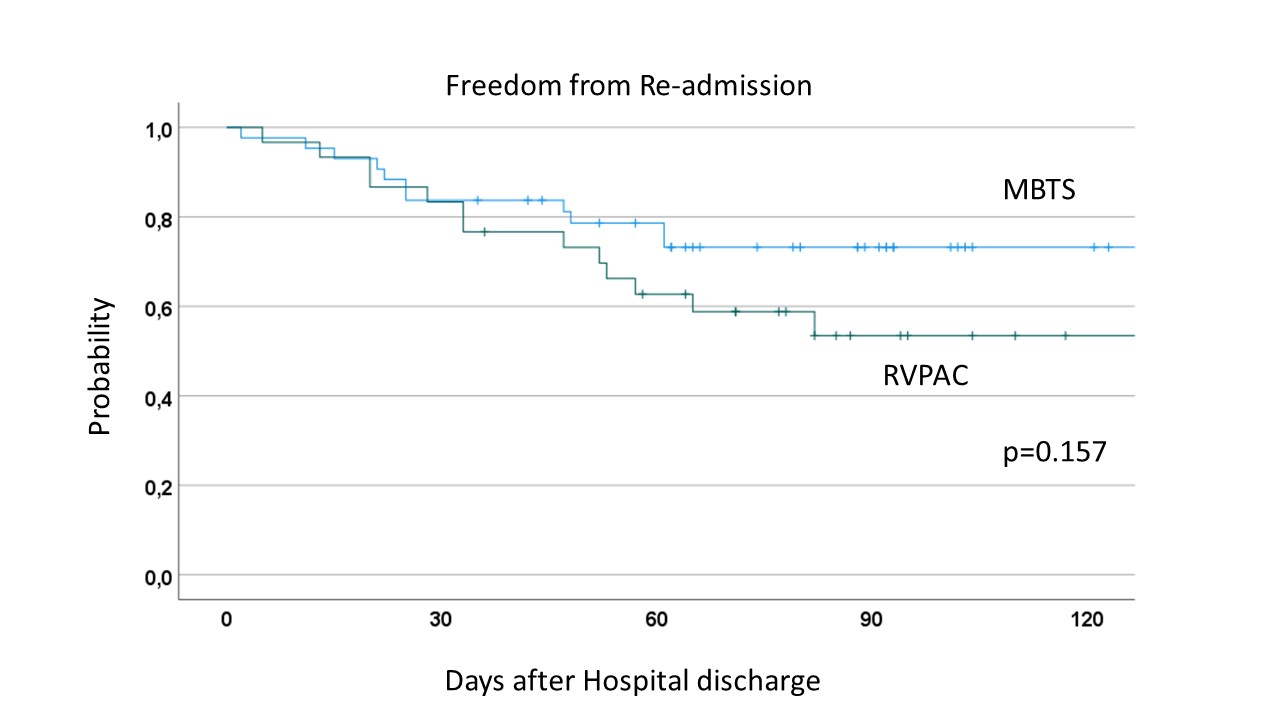
**
