# Supplementary figures and images for: Impact of home monitoring program on interstage mortality after the Norwood procedure
Source: Front Cardiovasc Med. 2023 Oct 11;10:1239477. doi: 10.3389/fcvm.2023.1239477 (PMC10600023; doi:10.3389/fcvm.2023.1239477)

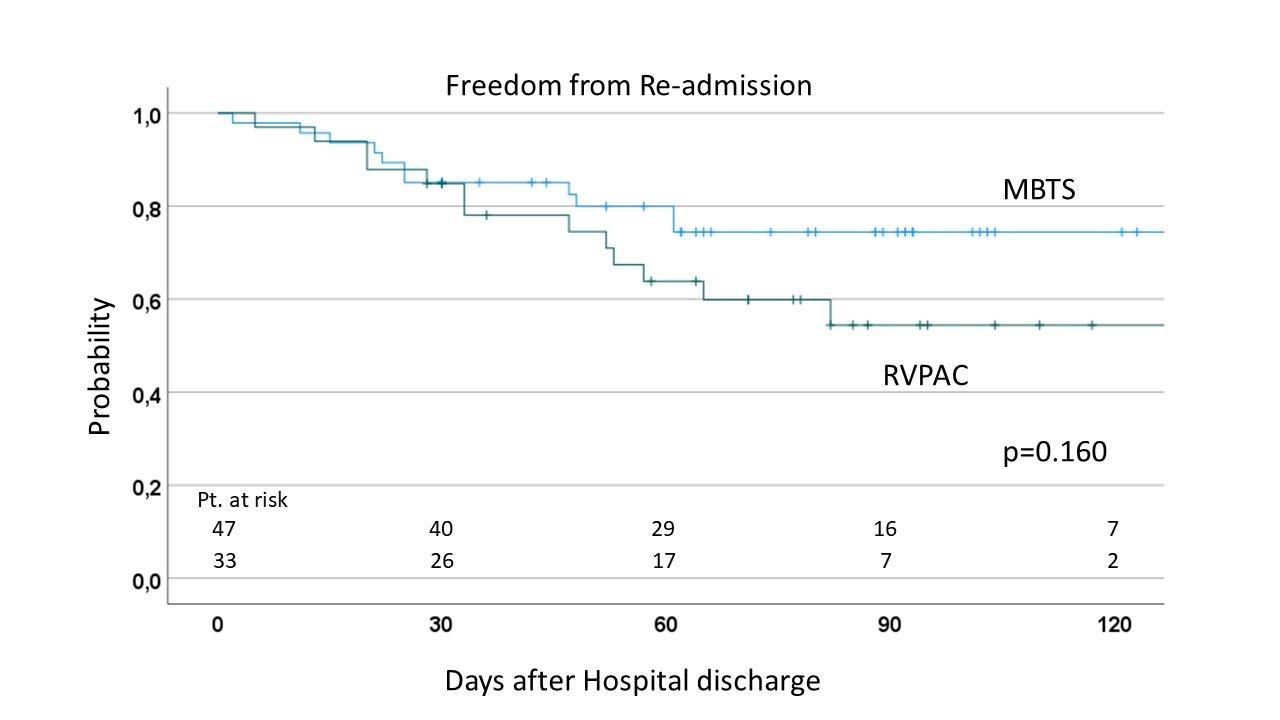

Supplement: Supplementary Figure S1 — Freedom from readmission after hospital discharge in patients with MBTS and with RVPAC. [file Image1.jpeg]
